# Risk of UTI in kidney stone formers: a matched-cohort study over a median follow-up of 19 years

**DOI:** 10.1007/s00345-020-03564-7

**Published:** 2021-01-05

**Authors:** Eleanor Brain, Robert M. Geraghty, Paul Cook, Paul Roderick, Bhaskar Somani

**Affiliations:** 1 Newcastle University Medical School, Newcastle-upon-Tyne, UK; 2 Department of Urology, Freeman Hospital, Newcastle-upon-Tyne, UK; 3 Department of Biochemistry, University Hospital Southampton, Southampton, UK; 4 Department of Public Health, University of Southampton, Southampton, UK; 5 Department of Urology, University Hospital Southampton, Southampton, UK

**Keywords:** Kidney stones, UTI, Urolithiasis, Urinary tract infection

## Abstract

**Purpose:**

To describe risk of UTI in Stone formers comparing to non-stone formers.

**Methods:**

Retrospective cohort study using electronic records for patients across southern England. Stone formers referred to a tertiary referral centre in Southern England, comparator patients were age and sex matched with 3:1 ratio from same database. Those with no documentation were excluded. UTI defined using ICD-10 codes. Risk of UTI presented as hazard ratio with 95% confidence interval, generated using cox regression. Sample size calculated using 80% power and significance set at 0.05.

**Results:**

Eight hundred and nineteen stone formers were included after 1000 records were screened for inclusion, with 2477 age and sex matched non-stone formers extracted from the same database. Sample size was calculated at 287 per group. Stone formers were at significantly increased risk of developing a UTI (HR 5.67; 95% CI 4.52–7.18, *p* < 0.001). Median follow-up was 19 years (IQR: 15–22).

**Conclusions:**

Kidney stone formers are at increased risk of developing urinary tract infections.

## Introduction

Kidney stone disease (KSD) has a high economic burden [1, 2] and is increasing in prevalence [3]. Multiple factors can contribute to stone formation, including the presence of bacteria in the urinary tract [4]. Urinary Tract Infections (UTIs) with urease-producing bacteria are known to contribute to the formation of struvite (magnesium-ammonium-phosphate) stones [5]. However, struvite stones are uncommon, accounting for around 4% of stones [6].

Association between bacteria and the development of the more common calcium oxalate and calcium phosphate stones remains unclear [4]. Over the past few decades evidence has emerged in support of a link [4]. Several studies have isolated bacteria from calcium oxalate and calcium phosphate stones [7–9]. Furthermore, sequencing of material from five stones (four of which were calcium oxalate) detected DNA from multiple bacterial strains [10]. It is unclear whether the presence of bacteria here is causal or coincidental [4].

There has been no large cohort study to date demonstrating the relationship between KSD and UTI at a wider population level. Our aim therefore was to describe the risk of UTI in stone formers in a cohort study. Our secondary aim was to compare risk of UTI with differing stone composition.

## Methods and materials

### Study population

The cohort consisted of patients with kidney stone disease (KSD) presenting to a tertiary referral hospital referred for metabolic assessment between 1990 and 2007. The population has been described in a previous cross-sectional study [11]. During this period, stone formers were routinely referred to this clinic by the urology team (both in Southampton and around the region—Dorset, Wiltshire and Hampshire) and general practitioners. 1000 (from 2801) patients were selected by block randomization after alphabetization of surnames.

Further information on past medical history and subsequent stone recurrence was ascertained retrospectively using hospital and general practice electronic records. The general practice electronic records is downloaded to the Care and Health Information Exchange (CHIE), a large database including data from 172 general practices within Hampshire and the Isle of Wight (95% coverage).

Retrospective data collected from CHIE included: age, sex, past medical history at first presentation including diabetes mellitus (see Table 1) and urinary tract infections (UTI). Subsequent stone episodes and stone type were ascertained using a combination of CHIE and hospital records.

**Table 1:** Patient Demographics

		Patients with KSD	Comparators
Age at presentation in years, mean (SD)		49 (14)	49 (14)
Sex, *n* (%)	Female	247 (29.1%)	741 (29.1%)
	Male	601 (70.9%)	1803 (70.9%)
Follow-up in years, mean (SD)		19 (5)	19 (5)
Primary stone composition, *n* (%)	Ca Ox	425 (50.1%)	–
	Urate	21 (2.5%)	–
	Ca Po	17 (2.0%)	–
	Struvite	5 (0.6%)	–
	Unclear	380 (44.8%)	–

**Fig. 1:**
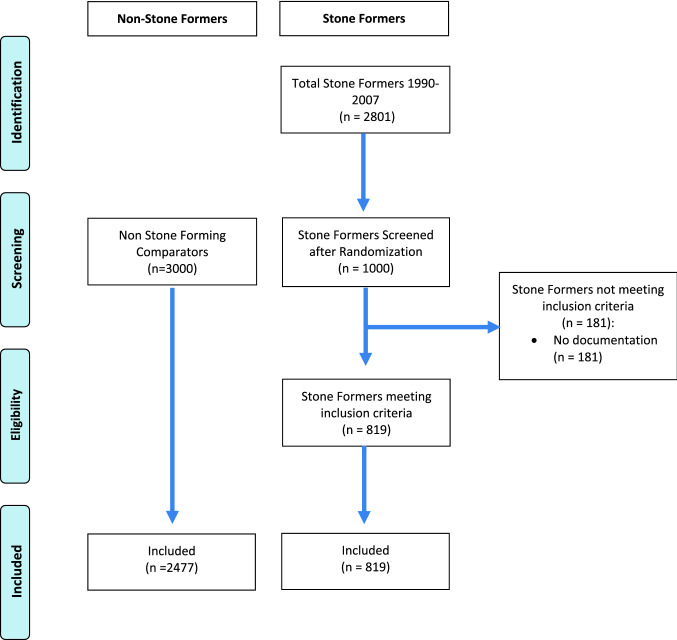
CONSORT Flow diagram of patient selection

Patients who had no documentation (i.e. no evidence of subsequent follow-up/consultation, lived outside/have left Hampshire or no documentation on CHIE) were excluded (see Fig. 1).

### Comparator population

Comparator data was supplied by Care and Health Information Analytics (CHIA), the body utilising CHIE data for research, using age, sex and region matched patients, in a ratio of 3:1, once stone formers (SF) had been screened for eligibility. This ratio was selected to increase power. Patients with codes associated with KSD were excluded.

### Definitions

Urinary tract infection (UTI) was defined as a documented symptomatic UTI on CHIE after time of first stone episode. As these are general practice records the diagnosis of ‘symptomatic UTI’ was based on symptoms, although it is unclear on whether it was based solely on symptoms, positive urine dip, positive urine culture or any combination of the above.

Diabetes mellitus defined as HbA1c > 48 mmol/mol (6.5%) or random plasma glucose more than 11 mmol/L using the National Institute for clinical and Health Excellence (NICE) guidelines [12], which are based on the WHO guidelines [13].

Stone recurrence defined as subsequent stone episode after previously being rendered stone free i.e. no stones.

### Statistical methods

SPSS version 26 and R statistical package version 3.6.3 (packages: survival and survminer) were used for statistical analysis. Cox proportional hazards model was used to analyse the data, which is presented as hazard ratio (HR) with 95% confidence interval (CI). Time to event was defined as time from presentation to metabolic stone clinic to development of UTI for both stone formers and comparators. Censoring time was defined as time from presentation to metabolic stone clinic to last CHIE entry or death.

Adjustments were made for presence of diabetes at any time during follow-up. Sub-analysis was conducted for stone type. The proportional hazards assumptions were tested by calculating Schoenfield residuals and performing a log-rank test.

Further analysis was performed for risk of recurrence in those who developed UTI. Time to event was defined as time from initial stone episode to development of second stone episode, where previously rendered stone free (no stones). Censoring time as defined above.

### Sample size calculation

Sample size was calculated estimating a 5% difference (20% in Stone formers, 15% in normal population) in rates of UTI diagnosis between the two groups. Power was set at 80% and significance at 0.05. Sample size was therefore calculated at *n* = 589 for the stone former group, and *n* = 1767 for the control group. Larger numbers have been included to increase power for subanalyses and ensure minimal loss to follow-up.

### Ethical approval

Ethical approval for this study was granted by the NHS Bristol Research Ethics Committee (Rec ref: 18/SW/0185; IRAS ID: 240061).

## Results

### Participant demographics

819 patients with KSD and 2477 stone free comparators were included, and were age and sex matched. The male:female ratio was 3:1. The mean age at first metabolic stone clinic presentation was 49 ± 14 years, with median follow-up of 19 years (IQR: 15–22). 155 stone formers (18.7%) developed at least one UTI during the study period, compared to 422 (14.1%) of the comparator population. Stone composition in the patients with KSD is described in Table 1. Deaths were similarly proportioned in the two groups with 113 (13.3%) amongst stone formers, and 366 (14.4%) amongst the comparators.

Of those 155 stone formers who developed a UTI, 63 had at least one stone recurrence. Of these, 16 patients had an associated UTI. There were 3 patients who developed urosepsis following their initial stone presentation, however these were not associated with a stone episode. Comparing GP records and hospital records of those who had UTIs associated with recurrence, demonstrated that 5 had a coded UTI on the GP record within one month of a hospital episode, and that 6 had a coded UTI at exactly the same date as the hospital record for a stone episode.

### Risk of UTI in patients with KSD

**Fig. 2:**
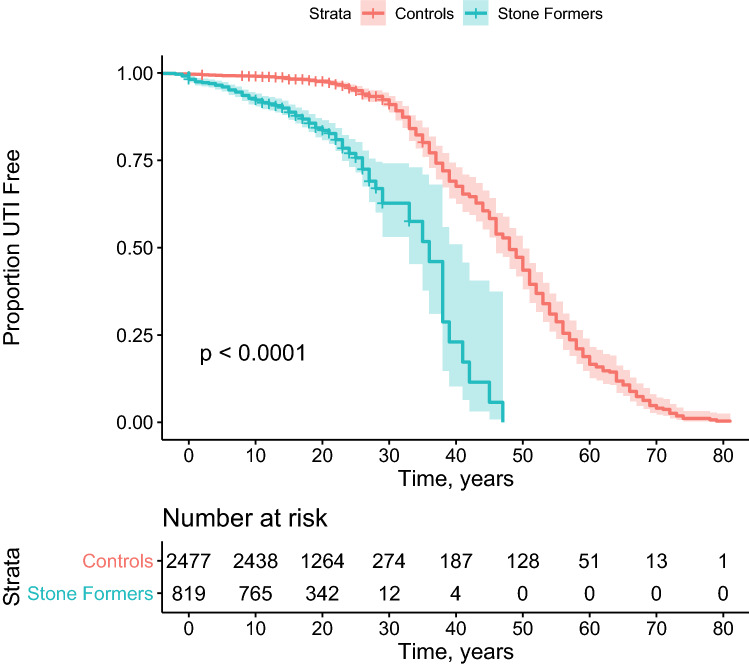
Kaplan Meier curve for time to development of UTI

Patients with KSD had a significantly increased risk of UTI (HR 5.73; 95% CI 4.55–7.21, *p* < 0.001). This effect was robust to adjustment for diabetes mellitus (HR 5.76 95% CI 4.50–7.36, *p* < 0.001) (Fig. 2).

Subanalysis by stone composition demonstrated significantly higher risk of UTI in patients with calcium oxalate stones (HR 6.36; 95% CI 4.82–8.40, *p* < 0.001) and urate stones (HR 6.87; 95% CI 2.82–16.72, *p* < 0.001) (Table 2). Participants with other stone compositions did not have significant risk of UTI.

**Table 2:** Subanalysis of risk of UTI per stone composition

Stone composition	HR (95% CI)	*p*
CaOx	6.36 (4.82–8.40)	< 0.001
CaPo	1.88 (0.26–13.43)	0.53
Urate	6.87 (2.82–16.72)	< 0.001
Struvite	6.80 (0.95–48.71)	0.056
Unclear	5.17 (3.85–6.94)	< 0.001

Stone formers with subsequent UTIs were not at increased risk of a stone recurrence (HR: 0.96; 95% CI 0.70–1.33, *p* = 0.82).

**Fig. 3:**
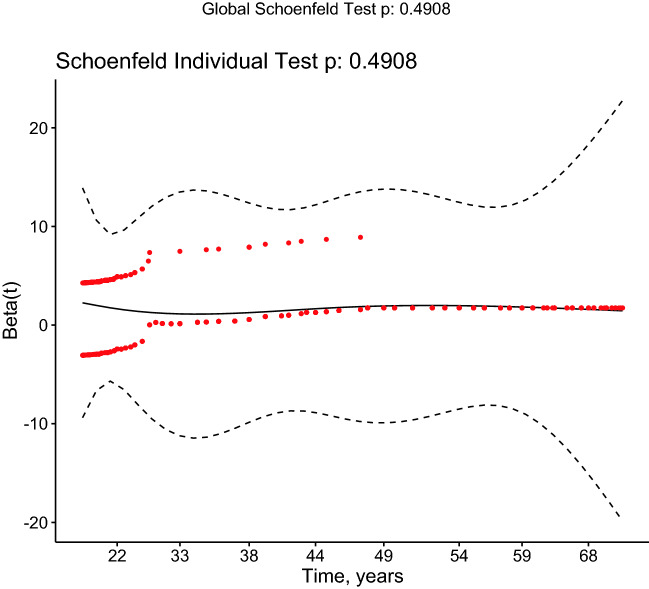
Schoenfeld residuals plotted against time. Loess line with 95% CI

Log rank demonstrated a significant result (*p* < 0.001). Visual inspection of the Schoenfeld residuals did not demonstrate variation around 0, and was non-significant (global Schoenfeld test, *p* = 0.491) (see Fig. 3).

## Discussion

Previous studies support bacterial involvement in the development of urinary stones, but this is the first large cohort study to examine the risk of UTI in patients with KSD. There was a significantly increased risk of UTI in patients with KSD in this population; this effect was robust to adjustment for diabetes mellitus. Moreover, those stone formers who developed UTIs were not at increased risk of recurrence.

The strengths of this study include suitable power to detect the primary outcome along with age- and sex-matching. Weaknesses include the possibility of referral bias, although our population is likely to be representative as the recurrence rate is similar to previously reported cohorts[14] and proportions of stone type are also similar[15]. Those lost to follow-up were included as far as practicable but those with no further data on contact with healthcare professionals were excluded as it was not possible to ascertain whether these patients developed UTIs. Missing data and potential inaccuracy of coding is another weakness; this is particularly relevant for electronic data in primary care which can be more variable in accuracy than secondary care [16]. The data for patients with KSD and comparators were from the same dataset however, so any missing or inaccurate data will be inherent to both populations.

The sub-analyses are likely limited by lack of power, especially urate, calcium phosphate and struvite stones. Although the former two are significant, the latter is not. One would expect struvite stone formers to have a significant risk of UTI given their infectious aetiology [5]. Likewise the non-significant result for no difference in recurrence in those stone formers who did, or did not, get a subsequent UTI may be due to lack of power. Those with an unclear stone type are to be expected in a general stone forming population, as some may pass their stone spontaneously. Rates of up to 85% unknown stone types are seen in the literature[17], compared to our study that has around 40%.

Our observation that patients with KSD are at increased risk of developing UTI compared to a comparator population corresponds with the previous observation by Holmgren et al. that 28% of patients admitted to a Swedish hospital with KSD over 7 years had a positive urine culture [18]. A further study has demonstrated that successfully treating a stone can lead to resolution of UTIs [19]. These findings not only highlight the clinical association between KSD and UTI, but also support previous evidence suggesting a mechanistic association between the two through detection of bacteria within the stones themselves [7–10]. However, the nature of such a link that may underlie our observations remains unclear, so should be the subject of future studies. Some potential mechanisms have been postulated based on initial investigations [4]. Bacteria appear to selectively attach to crystals and may increase aggregation of crystals [9, 10, 20]. The presence of bacteria has also been shown to increase expression of stone matrix proteins which could trigger progression to stone formation [4]. It is therefore plausible that urinary stones may act as a foreign body, becoming colonised in the same way that a urinary catheter is invariably colonised. As only a small proportion of patients with colonised catheters become symptomatic, it is reasonable to assume that the same applies to colonised urinary stones, with only a subset of patients developing symptomatic UTIs.

Further study is needed to fully establish the relationship between UTIs and kidney stones. Urologists and other practitioners should be mindful that stone formers are at increased risk of UTIs. From a global health perspective this increased risk along with the increasing prevalence of stone formation in the wider population will lead to an increased burden of UTIs.

## Conclusion

Patients with KSD are at increased risk of developing UTIs. Subanalysis of stone type demonstrated significantly increased risk of UTI in those developing calcium oxalate and urate stones. This has implications for future management of these patients.
